# The Human Microbiota and Skin Cancer

**DOI:** 10.3390/ijms23031813

**Published:** 2022-02-05

**Authors:** Yu Ri Woo, Sang Hyun Cho, Jeong Deuk Lee, Hei Sung Kim

**Affiliations:** Department of Dermatology, Incheon St. Mary’s Hospital, The Catholic University of Korea, Seoul 06591, Korea; w1206@naver.com (Y.R.W.); drchos@yahoo.co.kr (S.H.C.); leejd@catholic.ac.kr (J.D.L.)

**Keywords:** human microbiota, skin microbiota, non-melanoma skin cancer, melanoma

## Abstract

Skin cancer is the most common type of cancer in the US with an increasing prevalence worldwide. While ultraviolet (UV) radiation is a well-known risk factor, there is emerging evidence that the microbiota may also contribute. In recent years, the human microbiota has become a topic of great interest, and its association with inflammatory skin diseases (i.e., atopic dermatitis, acne, rosacea) has been explored. Little is known of the role of microbiota in skin cancer, but with the recognized link between microbial dysbiosis and inflammation, and knowledge that microbiota modulates the effect of UV-induced immunosuppression, theories connecting the two have surfaced. In this paper, we provide a comprehensive review of the key literature on human microbiota, especially the skin microbiota, and skin cancer (i.e., non-melanoma skin cancer, melanoma, cutaneous T cell lymphoma). Also, mechanistic perspectives as to how our microbiota influence skin cancer development and treatment are offered.

## 1. Introduction

Skin cancer, which encompasses melanoma and non-melanoma skin cancer (NMSC), represents the most common form of malignancy in Caucasians and its global burden continues to rise [1,2]. The pathogenesis of skin cancer is multifactorial, and while there are well-characterized environmental risk factors such as ultraviolet (UV) radiation, many remain undetermined.

The human body is colonized by a vast number of microbes, collectively referred to as the human microbiota. The link between these microbes and our health is the focus of a growing number of research initiatives, and new insights are emerging rapidly.

The cancer microenvironment, which denotes non-cancerous cells (i.e., endothelial cells, fibroblasts, immune cells) within the tumor, is recognized to modulate cancer progression and response to treatment. Chronic inflammation is a hallmark of cancer where evidence suggests that it creates a pro-cancer microenvironment.

Given the emphasis on microbial composition and its involvement in human disease in recent years, the question arises as to how an individual’s distinct microbiota, which outnumber human cells by a factor of 10 [3], may influence skin cancer risk and subsequent response to therapy. Since microbial dysbiosis is linked with chronic inflammation, inflammation-mediated carcinogenesis processes, and immune evasion, it is not surprising that microbes are associated with the development of specific cancers. Such relationship has been reported with the role of *H. pylori* in gastric cancer and *Fusobacterium* in colorectal cancers. However, relatively little is known of the connection between the human microbiota and skin cancer.

In this review, we discuss the human microbiota (with a focus on the skin microbiota) that are most closely linked with skin cancer and explore their mechanism of action and therapeutic potential.

## 2. The Skin Microbiota and Skin Cancer

### 2.1. Non-Melanoma Skin Cancer (NMSC)

NMSC is the most common cancer worldwide with an increasing incidence. The NMSC is often regarded as all cutaneous malignant neoplasms not associated with melanocytes. The squamous cell carcinoma (SCC) and basal cell carcinoma (BCC) compromise up to 99% of NMSC [4].

Kullander et al. [5] found the association between *Staphylococcus aureus* (*S. aureus)* and SCC by looking into tumor biopsies and swab samples, respectively. The colonization of *S. aureus* was higher in SCC biopsies (29.3%) than healthy skin biopsies (5.7%). In addition, the prevalence of *S. aureus* in swab samples from SCC (31.7%) was higher than healthy skin swab samples (15.0%). They found that an increased prevalence of *S. aureus* DNA was strongly associated with actinic keratosis (AK) and SCC of the skin by targeting *S. aureus* specific *nuc* gene in their polymerase chain reaction (PCR)-based experiment [5]. As AK is a precancerous lesion of SCC, increased colonization of *S. aureus* in AK implies that *S. aureus* is associated with the carcinogenic process of AK to SCC [5]. Consistent with findings from the prior studies, Wood et al. [6] also reported that *S. aureus* is the most abundant bacterial species in the lesional skin of AKs and SCCs by swab sampling. A recent study has also found that *S.* *aureus* is overabundant in AK and SCCs by 16S rRNA gene-based microbial community profiling with samples from skin biopsies [7]. The increased abundance of *S. aureus* was also associated with an increased expression of human beta defensin-2 (hBD-2) in SCCs [7]. In a co-culture study using *S. aureus*, SCC cell lines from cutaneous SCCs, and Hecate cells, the expression of hBD-2 was increased by *S. aureus* challenge, along with an increased cellular proliferation of tumor cells [7]. Moreover, the challenge of SCC cells directly with hBD-2 resulted in an increased proliferation of tumor cells [7]. This study suggests that the overabundance of *S. aureus* in SCC can affect the expression of hBD-2, which might induce the proliferation of SCC. Although *Staphylococcus* genus comprises many commensal species on human skin with a number of studies identifying overabundance of *S. aureus* in SCCs, there is lack of evidence on the causal association between *S. aureus* with SCCs. The overabundance of *S. aureus* is not well observed in hyperkeratotic skin tumors including seborrheic keratosis and psoriasis [5,7]. Indeed, *S. aureus* does not infect an immunocompromised individual when the skin barrier is not disrupted [8]. However, under certain conditions including burn scars and atopic dermatitis where the skin barrier is obviously disrupted, over-colonization of *S. aureus* is frequently observed [9,10]. These findings suggest that barrier disruption in SCC is important for the colonization of *S. aureus*.

A cohort study conducted in Australia showed that the abundance of *Cutibacterium* in the skin of AK and SCCs is decreased compared to photodamaged non-lesional healthy skin. *Cutibacterium* is a genus of commonly observed skin-resident bacteria with a lipophilic property [6]. Their common habitats are well known as sebaceous areas of the skin. It has been suggested that decreased abundance of *Cutibacterium* in AK and SCC could be caused by the dry and scaly surface of AKs, which might be associated with decreased availability of sebum [6]. The decreased abundance of these taxa reflects a decrease in humidity and an elevated pH of the skin. In addition, since *Cutibacterium acnes* *(C. acnes)* can produce AMPs, decreased abundance of *Cutibacterium* might be associated with an imbalance of microorganisms of the skin of AKs and SCCs [6]. We suppose that altered metabolism in tumor cells might inhibit the growth of lipophilic commensals such as *Cutibacterium* and induce the growth of *S. aureus*.

*Staphylococcus epidermidis (S. epidermidis)* can exert a protective effect against the development of skin tumors. *S. epidermidis* is a frequently observed coagulase-negative staphylococcal species that resides in healthy human skin [11], and its existence inhibits the growth of *S. aureus* [12]. Cell-free conditioned media from *S. epidermidis* can suppress biofilm formation of *S. aureus* through an *icaR-*dependent pathway and the *Rsp* gene, which is an AraC-type transcriptional regulator prohibiting attachment and biofilm formation in *S. aureus* [12]. In addition, phenol-soluble modulins (PSM)γ and PSMδ from *S. epidermidis* can exert an antimicrobial action against cutaneous bacterial pathogens such as *S. aureus* and *group A streptococcus* [13,14]. It has been confirmed that the secretome of *S. epidermidis* can suppress skin inflammation by activating regulatory T cells [15]. A recent study by Nakatsuji et al. [11] found that 6-N-hydroxyaminopurin (6-HAP) produced by *S. epidermidis* can suppress the synthesis of DNA and exert an antiproliferative effect on transformed tumor cells. In a mouse model, colonization of a strain of *S. epidermidis* capable of releasing 6-HAP was shown to decrease the incidence of UV-induced tumor formation compared to that of a control strain that does not release 6-HAP. Among human commensal strains of *S. epidermidis,* the metagenomic sequence analysis also found the MO34 strain of *S. epidermidis*, a common identifiable strain similar to 6-HAP releasing strain in mice, in their analysis [11].

With regards to fungi, decreased colonization of *Malassezia* in the skin of AK and SCCs compared to non-lesional healthy skin has been observed [6,7]. *Malassezia* is a well-known lipophilic skin-resident commensal. Skin barrier disruption and decreased sebum availability might explain the decreased colonization of *Malassezia* in SCCs. Recently, it has been reported that *Malassezia* suppresses the *S. aureus* biofilm formation by secreting specific proteases [16]. Based on findings, we can assume that *Malassezia* has a protective role against *S. aureus* colonization in SCC.

An epidemiological study by Chung et al. [17] has found that *Candida* infection is associated with increased risk of various cancers including hematologic, head and neck, pancreatic, skin, and thyroid cancer in a nationwide population-based study. However, experimental studies directly identifying the link between fungal microbiota and skin cancer are scarce. Therefore, studies identifying the role of fungal microbiota in the development of NMSC should be conducted in the future.

Emerging studies have shown a possible link between SCC and human papillomavirus (HPV). A recent population-based study conducted in Taiwan has found an increased risk of HPV infection in patients with NMSC (adjusted hazard ratio, 2.59; 95% confidence interval [CI], 1.43–1.71) compared with controls [18]. A meta-analysis also found an increased risk of HPV infection in cutaneous SCC than in normal-appearing skin (pooled effect size, 3.43; 95% CI, 1.97–5.98) [19]. Moreover, an increased prevalence of HPV was observed in immunocompromised patients than in immunocompetent patients (pooled effect size, 3.01; 95% CI, 2.00–4.52) [19]. The HPV family constitutes about 200 subclasses that can affect the mucosal and skin epithelium [20]. E6 and E7 are pivotal oncoproteins involved in various cellular processes such as the cell cycle, apoptosis, DNA repair, and senescence [21]. To date, about 50 beta HPV types have been identified, where beta HPV types are mainly associated with cutaneous SCC. Beta HPV could be classified into five subclasses: beta-1, beta-2, beta-3, beta-4, and beta-5 [22]. A synergistic effect between cutaneous beta HPV types and ultraviolet radiation (UVR) might play a pivotal role in the rise of cutaneous SCC. Beta HPV types such as HPV5 and HPV8 have been isolated from the skin of patients with epidermodysplasia verruciformis, which commonly progresses to cutaneous SCC [23]. In a mouse model study, transgenic mice expressing beta HPV type E6/E7 genes and placed under UV irradiation showed increased development of SCCs [24,25,26]. Of interest, the expression of E6 and E7 from various beta HPV infections in cooperation with UV irradiation specifically acts on the initial phase of skin carcinogenesis without being involved in the maintenance phase. Viarisio et al. [27] have also reported that chronic UV irradiation to transgenic mice with beta HPV38 E6 and E7 oncogenes can increase UV-induced mutations. The pattern of mutation in the skin of transgenic mics is similar to that detected in human NMSC, with increased rates of mutations in p53 and Notch genes. However, after the development of UV-induced skin lesions, deletion of viral oncogenes using the Cre-lox recombination system did not alter the growth of the tumor, implying that beta HPV types are only involved in the initial phase of carcinogenesis [27]. In fact, a study was conducted on three human cutaneous SCCs using 454 high-throughput pyrosequencing to find novel viral sequences in cutaneous SCC but failed to find transcripts of oncogenic human viruses [28]. Although transcripts of the virus with very low abundance or low sequence homology to already well-known oncogenic viruses might exist, the authors have suggested that HPVs are not transcribed in most SCC or necessary for the maintenance of SCC [28]. The hypothetical mechanism of action of beta HPVs in cutaneous carcinogenesis could be explained by the hit-and-run mechanism [27]. Cutaneous beta HPVs can act as cocarcinogens to promote cellular damage under UV irradiation. However, they are not needed for the maintenance of SCCs. Strickley et al. [29] recently found a more significant decrease in the viral load and activity in human skin biopsy samples than in perilesional skin samples using RNA and DNA in situ hybridization for 25 commensal β-HPVs. The authors suggested the existence of selective immunity against virus-positive tumor cells as T cell immunity against commensal HPVs can inhibit tumorigenesis in immunocompetent individuals, while the loss of T cell immunity is associated with an increased risk of skin cancer in immunosuppressed individuals [30]. Given the diversity regarding the role of HPVs in skin cancer development, further studies are needed to confirm this association.

The presence of Merkel cell polyomavirus (MCPyV), a recently identified human virus, is involved in the pathogenesis of Merkel cell carcinoma (MCC). The presence of MCPyV is also associated with high tumor burden of MCC [30]. With regards to SCC, MCPyV was detected in 15% of DNA samples of immunocompetent SCC patients [31]. Despite the detection rate of MCPyV in SCC, a possible link between MCPyV and SCC needs further elucidation.

### 2.2. Malignant Melanoma

Among various skin cancers, malignant melanoma (MM) is the most lethal form of skin cancer, comprising 75% of all skin cancer-associated deaths [32]. The incidence of MM differs worldwide with the highest incidence observed in Australia (50.3/100,000) and New Zealand (47.4/100,000) [33]. The incidence and mortality of MM have steadily increased over the last 40 years. MM is a heterogeneous disease with various subtypes based on its anatomical distribution, pattern of somatic mutation, and histopathological feature [32]. Although the involvement of the gut microbiota in MM has received much attention recently, few studies have been conducted to identify the role of the skin microbiota in patients with MM. A recent culture-based microbial analysis conducted on 27 patients with acral melanoma by skin swabs found that the genus *Corynebacterium* is more strongly associated with stage III/IV MM patients compared to stage I/II MM patients [34]. A higher number of interleukin (IL)-17 positive cells were found in the genus *Corynebacterium*-positive patients than in genus *Corynebacterium*-negative patients [34]. IL-17 can induce the growth of melanoma by upregulating IL-6 and the signal transducer and activator of transcription 3 [35]. In a mouse model study, application of *Corynebacterium accolens* suspension resulted in an increased infiltration of γδ TCR positive IL-17A-producing T cells on the dermal skin [36]. Taken together, these findings suggest that *Corynebacterium* species might affect the development of MM through an IL-17 dependent pathway. Another mouse study showed intra-tumoral injection of *C. acnes* to significantly decrease tumor size [37]. After intra-tumoral injection with *C. acnes,* the growth of melanoma cells was inhibited through the induction of Th1 type cytokines such as IL-12, tumor necrosis factor alpha (TNF-α), and interferon gamma (IFN-γ) [37].

Nakatsuji et al. [11] suggested that intravenous injection of 6-HAP derived from *S. epidermidis* can inhibit the growth of B16F10 melanoma cell lines, implying a protective role of *S. epidermidis* against MM. On the contrary, Wang et al. [38] suggested that *S. epidermidis* and lipoteichoic acid (LTA) from *S. epidermidis* can enhance the survival of melanocytes by upregulating TRAF1, CASP14, CASP5, and TP73 during UVB irradiation. They also reported that *C. acnes* can suppress the survival of UVB-irradiated melanocytes by promoting apoptosis, enhancing the secretion of coproporphyrins, and upregulating TNFα [38]. The exact role of *S. epidermidis* in the proliferation of melanocytes and the development of MM should be further elucidated. Salava et al. [39] used human skin samples to identify the human skin microbiome of melanocytic disorders including cutaneous melanoma and melanocytic nevi. Control samples were taken from normal skin of the contralateral body part within the same individual [39]. 16S ribosomal RNA sequencing revealed marginally decreased microbial diversity in melanoma skin samples than melanocytic nevi [40]. Mrazek et al. [40] also studied the skin microbiome in a melanoma model using Melanoma-bearing Libeechov Minipig. They found significant differences in bacterial compositions and microbial diversity between melanoma and normal skin samples [41]. The abundance of *Fusobacterium* and *Trueperella* were increased in melanoma skin samples than in controls [40]. In addition, the increased abundance of *Fusobacterium nucleatum* was associated with disease progression [40]. The association between *Fusobacterium* and various cancers including pancreatic, colorectal, and oral cancers has been found in previous studies [41,42,43]. Among *Fusobacterium, Fusobacterium nucleatum* may potentiate tumor proliferation by inhibiting the cytotoxicity of natural killer cells through interaction of Fusobacterial protein Fap2 and T cell immunoglobulin and ITIM domain (TIGIT) [44].

The role of virus in cutaneous melanoma is somewhat contradictory. Several epidemiological studies have suggested the association between HPVs and melanoma. A population-based cohort study demonstrated that a HPV infection is associated with an increased risk of melanoma (adjusted hazard ratio, 17.1; 95% CI, 1.88–156) [18]. As a matter of fact, high-risk mucosal HPVs have been found in 27% of MM samples (skin biopsy) using PCR-ELISA [45]. Among the various high-risk HPVs, HPV 16 and HPV 33 were highly detected [45]. A study on uveal melanoma has suggested that downregulation of HPV 18 E6/E7 can inhibit tumor growth and block the cell cycle by activating p53 and Rb pathway [46]. With regards to beta HPVs, HPV22 was more prevalent in melanoma than in normal control skin from the same individual [47]. However, the clinical and pathological characteristics of MM were not specifically linked with HPV prevalence [47]. Further studies should be performed to determine whether cutaneous HPVs could be a cofactor in the development of MM.

Regarding Merkel Cell Polyomavirus (MCPyV), a study found no association between MCPyV and melanoma [48]. However, Mokanszki et al. [30] identified four MCPyV positive cutaneous melanomas among 60 melanoma samples, while finding little association between MCPyV infection and melanoma burden [30]. To date, the pathogenic relationship between MCPyV and melanoma is unclear and warrants further investigation.

Human endogenous retroviruses (HERVs) can act as a cellular reservoir of pathogenic retroviral genes. Activation of ERV sequence is associated with melanocyte transformation and induces melanoma cells to escape from immune surveillance [49]. Increased expression of retroviral envelope protein and activation of retroviral *pol* gene have been observed in melanoma cell lines after UVB irradiation, suggesting the UVR-associated pathogenesis of melanoma [49].

### 2.3. Cutaneous T Cell Lymphoma

Cutaneous T cell lymphoma (CTCL) is the most common type of primary cutaneous lymphoma. It is an extranodal non-Hodgkin’s lymphoma characterized by malignant T cell accumulation confined to the skin. Among CTCLs, mycosis fungoides (MF) and Sezary syndrome (SS) constitute about 53% of cutaneous lymphoma [50]. Although early stages of MF show an indolent clinical course, advanced stages of MF or SS, traditionally described as a leukemic form of CTCL associated with erythroderma, have a more aggressive clinical course [51]. Although the immunopathogenesis of CTCL needs to be further elucidated, chronic antigen exposure is considered to exert a major role in the development of CTCL. In a genetically susceptible patient, chronic exposure to exogenous or endogenous antigenic stimuli can cause CTCL. As the skin is the body’s most outermost surface, skin microbiota could be one of the antigenic stimuli in CTCL.

Various studies have linked *S. aureus* and CTCL. Talpur et al. [52] examined the colonization of *S. aureus* in patients with MF and SS and found that 63% and 54% of patients had skin and nasal colonization of *S. aureus,* respectively [52]. Colonization of *S. aureus* was heaviest in erythrodermic SS while scant in MF without erythroderma [52]. Moreover, topical treatment with nasal mupirocin twice daily for consecutive days and oral antibiotics (dicloxacillin 250 mg four times a day or cefalexin 1 g daily for penicillin-allergic patients) for 4 weeks to eradicate *S. aureus* colonization resulted in clinical improvement in 58% of CTCL patients [52]. Eight patients with treatment-resistant CTCL were also found to achieve clinical improvement of skin lesions after treatment with intravenous and oral antibiotics [53]. Here in the study, the malignant T cells were decreased in lesional skin biopsy specimens after treatment [53]. Also, following antibiotic treatment, patterns of mRNA expression in CTCL changed, becoming more similar to those found in normal healthy skin. Of note, a clear inhibition of IL-2 signaling and STAT3 activation was observed in CTCL after antibiotic treatment [53].

Previous studies have reported the association of HLA-DR5 and DQB1*03 class II alleles with CTCL [54,55], suggesting a role of *S. aureus* superantigen in CTCL. Staphylococcal enterotoxin A (SEA) isolated from the CTCL lesions were found to induce the activation of STAT3 and IL-17 in immortalized and primary malignant T cells [56]. Jackow et al. [57] also found that patients with positive culture of *S. aureus* are carriers of enterotoxin genes. Among the 16 strains, six were found to have the same toxic shock syndrome toxin-1-positive clone characterized by electrophoretic type 41 [57]. In addition to *S. aureus,* bacterial pathogens such as β*-hemolytic streptococci, Enterobacteriaceae, Pseudomonas aeruginosa*, and *Enterococcus species* were predominant in CTCL lesions [58]. Another study also found that eschars in the skin lesions of 10 CTCL patients were all colonized by *Enterococcus faecalis* [59]. Harkins et al. [60] analyzed the skin microbiome of CTCL in 4 patients with MF, 2 patients with SS, and 10 healthy volunteers using metagenomics sequencing from skin swab samples to demonstrate an increase in the mean relative abundance of *Corynebacterium* species and a decrease in the mean relative abundance of *Cutibacterium* species in skin samples of a healthy volunteer compared to a patient with MF or SS, implying a bacterial shift [60]. Salava et al. [61] used 16S and whole genome shotgun sequencing to analyze the characteristics of the skin swab samples from 20 CTCL lesions and 20 contralateral normal-appearing skin. The most abundant organisms at the genus level were as follows: *Staphylococcus* (30%), *Corynebacterium* (22.3%), *Cutibacterium* (5%), and *Streptococcus* (3%). The most abundant genera from whole-genome shotgun sequencing were as follows: *Cutibacterium* (25%), *Corynebacterium* (19%), and *Staphylococcus* (18%). When these data were further analyzed with DESeq2, *S. argenteus* was found to be more abundant in CTCL lesions than in normal-appearing healthy skin [61]. As *S. argenteus* can induce a 4- to 6-fold increase in the level of alpha-hemolysin exotoxin compared to *S. aureus* [62], the authors have suggested the possible pathogenic role of *S. argenteus* in CTCL. Further large-scale studies are needed to determine whether these bacteria are associated with the pathogenesis or the clinical course of CTCL.

Previous studies have postulated the possible role of viruses such as human T cell lymphotropic virus (HTLV) [63], Epstein–Barr virus [64], and human herpesvirus 8 [65] in the pathogenesis of CTCL. However, studies have failed to identify the association between CTCL and viral risk factors [66]. So far, findings are inconsistent with regards to the viral and fungal etiology in CTCL [60]. Further studies are needed to determine specific roles of microbiota and antibiotic therapies in CTCL.

## 3. Proposed Mechanisms between the Skin Microbiota and Skin Cancer

**Figure 1:**
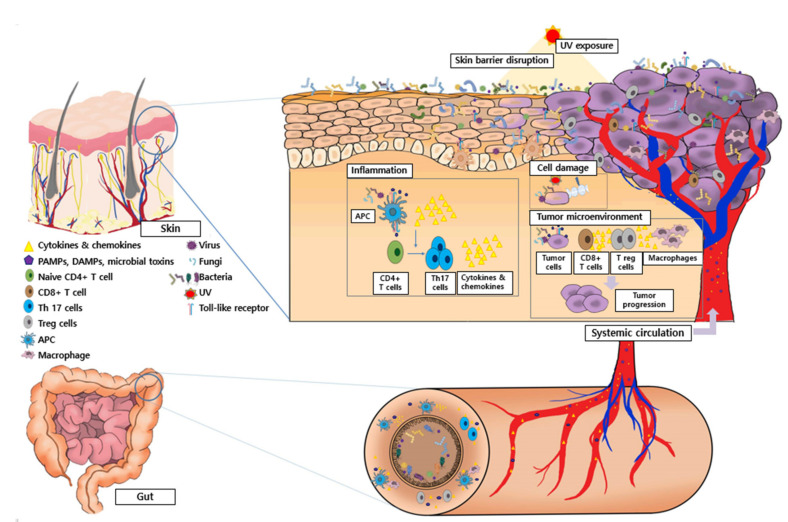
Proposed mechanisms between the skin and gut microbiota and skin cancer. A crosstalk between the disrupted skin barrier, UV exposure, and skin commensal microbiota can affect the composition of the skin microbiota. Altered skin microbiota along with damage-associated molecular patterns (DAMPs), pathogen-associated molecular patterns (PAMPs), and microbial toxins can induce chronic inflammation of the skin and cellular damage, which may lead to the initiation and progression of skin cancer. The microbiota, DAMPs, PAMPs, microbial toxins, CD8+ T cells, regulatory T cells, tumor-associated macrophages, and their corresponding cytokines and chemokines are major factors compromising the tumor microenvironment of skin cancers. They are involved in tumor progression by promoting immunosuppression, cellular proliferation, and inflammation in skin cancers. Additionally, microbial metabolites, cytokines, and chemokines from the gut can go through systemic circulation and impact the tumor microenvironment of the skin indirectly.

The primary habitat of the human commensal microbiota is known to be the gut. However, diverse microbial populations also reside in other parts of the body, such as the skin, oral cavity, respiratory tract, and genital tract. Among them, the skin is regarded as a pivotal ecosystem, as it is the largest body organ and a barrier to the external stimuli exerting immunological actions. The proposed mechanism of action between the skin and gut microbiota and skin cancer development is described in Figure 1.

### 3.1. The skin Immune System and Skin Cancer

The skin is the barrier between the internal and external environment and functions to maintain good health. The skin immune system is complex and consists of the innate and adaptive immune system. The pivotal function of the innate immune system is to defend against microbial pathogens until the adaptive immune system becomes activated. Major components of the innate immunity are keratinocytes, endothelial cells, fibroblasts, neutrophils, macrophages, dendritic cells, and mast cells, while T cells and B cells are known as adaptive immune cells [67]. Among them, keratinocytes are involved in innate immunity by producing various cytokines, chemokines, antimicrobial lipids, and antimicrobial peptides (AMPs) [68,69]. AMPs such as cathelicidin LL-37 and human β-defensin are constantly produced or upregulated under microbial stimuli such as pathogen-associated molecular patterns (PAMPs) or damage-associated molecular patterns (DAMPs) [70]. In fact, the commensal and pathogenic skin microbiota regulate the innate immunity of the skin. Pattern recognition receptors (PRRs) recognize unique molecular characteristics of PAMPs or DAMPs and induce an appropriate immune response. Various types of PRR are known according to the characteristic motif of the pathogen, location of intracellular expression, and signaling pathway, and are largely divided into cytoplasmic receptors and toll-like receptor (TLR)s. A total of 10 human TLRs have been identified to date [71]. Keratinocytes, melanocytes, and antigen-presenting cells in the skin can express TLRs. Recently, the association between various TLRs and skin cancer was extensively studied. Persistent activation of TLRs may promote chronic inflammation. Among various TLRs, TLR4 is known to play a pivotal role in both skin inflammation and cancer. Activation of TLR4 and subsequent internal signaling pathways can induce the activation of transcription factors including NF-κB, IRF-3, and AP-1, which affect the expression of genes associated with inflammation, cellular apoptosis, survival, and differentiation [72]. As a matter of fact, increased expression of TLRs has been observed in skin cancers. Overexpression of TLR4 has been observed in the skin of SCC when compared to control skin [73]. Recently, it has been found that the topical application of a TLR4 inhibitor, resatorvid, can reduce the size and number of tumors in a mouse model of UV-induced skin tumorigenesis, implying the benefit of blocking TLR in UV-induced NMSC [74]. In MM, the overexpression of TLR4 was observed in radial and vertical growth phases [75], where the expression of TLR4 was negatively associated with relapse-free survival [76]. A strong correlation between MCC and TLR4 expression has also been observed [77]. G100, a TLR4 agonist, has been shown to exert antitumor responses and enable tumor regression in patients with MCC [78]. Imiquimod, a TLR 7 agonist, is effective in managing various skin cancers such as SCC, BCC, CTCL, and lentigo MM [79]. Skin microbiome not only induces innate immune responses, but also activates the adaptive immune system in the skin, with downstream effects. Skin commensals also regulate the action of various effector T cells and IL-1 family signaling [80]. Moreover, recent evidence supports the importance of Th17 cell and its effector cytokines in explaining skin inflammation and cutaneous carcinogenesis by microbiota [81,82]. Although the paradoxical role of Th17 cells in the tumor microenvironment (TME) of MM has been raised, evidence supports the protumor effect of Th17 cells [83]. Further studies identifying the specific skin microbiota that enhance the response of Th17 cells in the skin are needed in the future.

### 3.2. Microbial Metabolites and Toxins in Skin Cancer

It has been suggested that all tumors revise their environment via paracrine pathways. The TME is composed of various immunocytes, fibroblasts, vascular and lymphatic endothelial cells, pericytes, adipocytes, and diverse secretable molecules by both the tumor and non-tumor cells [70,84]. In addition, the microbiome is now considered an important component of TME. Microbial metabolites can directly interplay with cancer cells or affect carcinogenesis by regulating other components of TME [85,86,87]. The proposed mechanism of action by how microbial metabolites affect cancer development includes modulation of the availability of metabolites, promotion of DNA damage, and modulation of the immune system [70]. Although little is known about the role of skin-derived microbiome metabolites in skin cancer, the effects of skin metabolites on a number of cutaneous inflammatory disorders such as atopic dermatitis and psoriasis have been well studied [88,89]. In patients with atopic dermatitis, alteration in skin microbiota-associated tryptophan metabolite, indole-3-aldehyde, has been observed [88]. The level of indole-3-aldehyde is negatively associated with the degree of skin inflammation in atopic dermatitis [88]. Besides metabolites, toxins produced by skin microbiota can alter the DNA and enhance the probability of oncogenic mutations [90]. For example, exotoxin from *S. argenteus* and Staphylococcus enterotoxin A have been associated with CTCL in previous studies [56,61]. However, to date, most studies focused on circulating metabolites and microbial toxins released from the gut microbiota. We suppose that the skin microbiota can also produce a wide variety of metabolites and related microbial toxins. Therefore, further studies are needed to identify relevant metabolites and toxins from the skin microbiota in patients with skin cancer.

### 3.3. Barrier Disruption in Skin Cancer

As the human skin functions as a primary outermost barrier between the host and its environment, a disrupted skin barrier might result in microbial dysbiosis [91]. Several studies have found that skin injury can alter the cutaneous homeostasis of the host and its commensal microbiota [91,92]. A barrier-deficient mouse model lacking three major proteins (envoplakin, periplakin, and involucrin (EPI)) was found to have similar bacterial phyla to the skin of wild-type mice [93]. However, the bacteria were three-fold more in the skin of EPI knockout mice than in the skin of wild-type mice [93]. In addition, a deeper penetration of bacteria was observed in EPI knockout mice than in the wild-type [93]. Moreover, increased expression of AMPs was observed in EPI knockout mice compared to the wild-type mice [93], suggesting that barrier disruption can lead to microbial dysbiosis. On the other hand, protease from the skin microbiota can result in epidermal barrier disruption. *Staphylococcus epidermidis* extracellular cysteine protease A was shown to damage the epidermal barrier in patients with Netherton syndrome [94]. With regards to skin cancer, skin barrier disruption in a NMSC mouse model has been observed in a study [59]. In addition, chronic inflammation in chronically injured or diseased skin can promote SCC [95]. Over-colonization of *S. aureus* has in fact been observed in SCC in various studies [5,6,7], with increased hBD expression and the growth of tumor cells [7]. However, microbiota specifically related to barrier disruption in skin cancer has not been elucidated yet. Whether barrier disruption alters the composition of the skin microbiota or whether alteration in the skin microbiota affects barrier disruption, thereby promoting carcinogenesis, needs further elucidation.

### 3.4. Ultraviolet Radiation and Skin Microbiota in Skin Cancer

Ultraviolet radiation (UVR) is considered a pivotal carcinogen in skin cancer. The effect of UV exposure on skin cancer is complicated and varies according to cancer types. Overall, photo-carcinogenesis occurs by UVR inducing DNA damage and progressive mutation, and causing clonal expansion of cancer cells, production of reactive oxygen species, and immune suppression. Several epidemiological studies have found that chronic UV exposure is a risk factor for skin cancer [96]. The high-intensity sunlight exposure was associated with the development of BCC [97]. With regards to SCC, UVR is classified as a pivotal carcinogen by International Agency for Research on Cancer. It is known to be involved in the initiation and progression of cutaneous SCC [98]. In addition, chronic UVR exposure is associated with melanoma, where UVR-induced DNA damage, inflammation, and immunosuppression results in the initiation, progression, and metastasis of primary cutaneous melanoma [99,100,101].

Bosman et al. [102] found that skin exposure to UVB can induce changes in the skin and gut microbiota. They suggested that UV-induced alteration of the microbiota had local and systemic effects [102]. Using germ-free mice, Patra et al. [103] found that UV-induced systemic immunosuppression is decreased in the presence of the skin microbiota. In addition, they found the degree of epidermal hyperplasia and infiltration of neutrophils were increased in the presence of microbiota, whereas infiltrations of mast cells, monocytes, and macrophages were increased in the absence of microbiota [103]. They also found significant differences in genetic expression according to the presence of the skin microbiota [103]. Increased expression of proinflammatory cytokine genes was observed in the presence of microbiota, whereas increased expression of immunosuppressive cytokine IL-10 was observed in the absence of microbiota [103]. Based on these findings, the authors suggested that skin microbiota can inhibit UV-induced immunosuppression by altering the gene expression of cytokines and cellular infiltration of the skin [103]. In another mouse study, colonization of 6-HAP-producing *S. epidermidis* decreased UV-induced skin tumors [11]. Weill et al. [104] also observed a protective action of a cell wall component of *Lactobacilli*, LTA, in a UV-irradiated hairless mouse model. Oral treatment with LTA resulted in a delay in tumor development and reversed UV-induced immune suppression [104]. The treated group showed increased levels of IFN-γ, helper, and cytotoxic T cells in the inguinal lymph nodes [59]. In addition, oral supplementation with LTA from *Lactobacillus rhamnosus* GG blocked UVB-induced immunosuppression and growth of skin tumors in another mouse model study [105].

### 3.5. Intratumoral Microbiota and Skin Cancer

Recently, the functional role of the intratumoral microenvironment in cancer biology was studied in various solid organ cancers. Nejman et al. [106] provided novel evidence that distinct tumors including melanoma, breast, pancreas, lung, ovary, brain, and bone tumors have a specific microbial composition. They also found that intratumoral bacteria are mostly present in the intracellular environment, involving both cancer cells and immune cells [106]. When MM patients responding to immune checkpoint inhibitor (ICI) were compared to non-responders, the ICI responders were found to have 18 more abundant and 28 less abundant taxa than ICI non-responders [106]. Among them, *Clostridium* was more abundant in responders, while *Gardnerella vaginalis* was more abundant in ICI non-responders [106]. To further determine how intratumoral microbiota affect the immune reaction of skin cancer, Kalaora et al. [107] sequenced 16S rRNA genes from 17 melanoma samples. They found the following seven genera to show greater abundance in melanoma: *Acinetobacter, Actinomyces, Comamonas, Corynebacterium, Enterobacter, Roseomonas,* and *Streptococcus* [107]. They demonstrated that: (1) bacteria that reside in melanoma can enter melanoma cells; (2) intratumoral bacterial HLA-I and HLA-II peptides can be presented by antigen-presenting cells; and (3) HLA-I and HLA-II molecules on melanoma cells can induce immune reaction [107]. Zhu et al. [108] used the RNA-Seq raw dataset retrieved from The Center Genome Atlas’s cutaneous melanoma dataset and found that decreased levels of CD8+ T cell infiltration are associated with a shorter survival. They further demonstrated the association between intratumoral bacteria and infiltration of CD8+ T cells [108]. Bacteria genus *Lachnoclostridium* showed to be the strongest positive correlation with infiltrating CD8+ T cells, followed by *Gelidibacter, Flammeovirga,* and *Acinetobacter,* whereas *Algibacter* and *Epilithnimonas* showed negative association with infiltrating CD8+ T cells [108]. In addition, intratumoral bacteria positively associated with infiltrating CD8+ T cells also induced increased expression of CXCL9, CXCL10, and CCL5 [108]. Intratumoral bacteria negatively associated with infiltrating CD8+ T cells were linked with decreased expression of these chemokines [108]. Bacterial load of *Lachnoclostridium* was significantly associated with a decreased risk of mortality, suggesting that increased infiltrating CD8+T cells and increased *Lachnoclstridium* abundance could be considered as good prognostic factors in patients with cutaneous melanoma [108]. As *Lachnoclostridium* is present in the gut microbiota in addition to intratumor tissues of cutaneous melanoma, a possible impact of the gut microbiome on the intratumoral environment through regulating CD8+ T cell infiltration has been suggested in their study [108]. Among various immune cells within tumor, Treg cells are well known to inhibit the anti-tumor immune response in various cancers, resulting in the immunosuppressive tumor microenvironment (TME). Infiltration of Treg cells is frequently observed in the skin samples of BCC, MM, and SCC [109,110,111]. It can inhibit actions of T cells and mediate immunosuppressive TME for skin cancers. The infiltration of type M2 tumor-associated macrophages was also observed in the TME and is thought to be involved in skin carcinogenesis. Further studies are needed to identify the complex interaction among intratumoral microbiome, tumor cells, and immune cells.

## 4. The gut Microbiota and Skin Cancer

The gut microbiota is the most widely studied among the human microbiota and has been linked with numerous inflammatory conditions. The gut microbiota serves as the largest endocrine organ by producing more than 30 hormone-like compounds, including glucagon-like peptide; leptin; cortisol; short chain fatty acids (SCFAs) such as propionate and butyrate; secondary bile acids; and several neurotransmitters such as serotonin, dopamine, tryptophan, and gamma-aminobutyric acid [112,113]. The gut and skin share similar properties, where the gut has a mucosal layer, which functions as the primary barrier between microorganisms and human cells in the GI tract [114]. In addition, the epithelium of the intestine consists of a single layer of enterocytes, and the barrier integrity is important in the maintenance of the immune system [115]. The commensal and pathogenic gut microbiota can use the intestinal mucus layer formed by mucin to enhance their growth, to form a biofilm, and to colonize in the intestine. The process of the immunological cascade is predominantly regulated by the interaction between endothelial cells and dendritic cells in mucosal tissues [116]. Disruption of the mucosal barrier usually induces dysbiosis of the microbiota and activation of host immune response.

Patients with cancer are frequently observed to have dysbiosis of the gut microbiota. The gut microbiota can interact with cancer cells via local or systemic effects and are associated with cancer development, progression, and response to cancer therapy. *Helicobacter pylori (H. pylori),* a well-known commensal and opportunistic pathogen in the stomach, is associated with the development of gastric mucosa-associated lymphoid tissue (MALT) lymphoma [117]. It has been suggested that chronic inflammation by *H. pylori* can induce malignant transformation of B cells [117], whereas eradication of *H. pylori* results in the regression of primary MALT lymphoma [118]. With regards to colorectal cancer (CRC), a Western diet enriched with animal protein and fat can induce the secretion of bile acid from the liver and enhance its delivery to the colon. Primary bile acids are usually secreted to the intestine. Through enterohepatic circulation, most of the primary bile acids are transported to the liver. Meanwhile, 5% of primary bile acids can avoid this circulation and become metabolized by the gut microbiota and transformed into secondary bile acids such as litocholic acid and deoxycholic acid (DCA) [119]. Patients with CRC show increased levels of bile acids in the colon. In addition, patients with multiple polypoid adenoma, a precursor of CRC, showed increased concentrations of DCA in their feces [120], implying that DCA might be associated with early stages of CRC. Certain gut bacterial species such as *Clostridium hylemonae* and *Clostridium hiranonis* can produce DCA, a genotoxic compound [119]. An enterotoxigenic *Bacteroides fragilis* was shown to produce DCA and induce Th17-mediated inflammation and colonic tumors in a mouse model [121].

Besides its association with cancers of the GI tract, the gut microbiota is also associated with cancer in other organs. For example, *Salmonella typhi* in the gut is associated with cancer in the gall bladder [122] and pancreas [123]. The intestinal microbiota can aggravate hepatocellular carcinoma by translocating lipopolysaccharide to the liver and activating TLR4 [124]. Concerning the skin, the gut microbiota can exert a significant effect on the skin and has been associated with various chronic inflammatory skin disorders, including acne, rosacea, atopic dermatitis, and psoriasis [125,126,127,128]. The proposed gut–skin axis suggests a possible link between the gut and skin microbiota. In patients with melanoma, responders to anti-programmed cell death (PD) 1 immunotherapy showed increased abundance of good bacteria in their gut compared to non-responders [129]. Authors also found that responders to PD-1 immunotherapy showed increased alpha diversity and higher abundance of *Ruminococcaceae* family than non-responders [129]. Routy et al. [130] also found that good responders to anti-PD1 immunotherapy among patients with epithelial tumors including NMSC, renal cell carcinoma, and urothelial carcinoma, have a greater abundance of *Akkermansia muciniphila*. Overall, the non-responders to immunotherapy have shown that gut dysbiosis and restoration of the gut microbiota altered the clinical response to cancer therapy. These findings suggest that the gut microbiota can be critical in regulating immunity against skin cancer. However, further research is needed to identify the association between gut dysbiosis and skin cancer.

## 5. Therapeutic Impact of Human Microbiota in Skin Cancer

Recently, the microbiota has been considered a crucial regulator of the tumor microenvironment by modulating the development and progression of the tumor and affecting treatment response. In accordance, prebiotics and/or probiotics are being considered promising therapeutic options with their ability to regulate skin and gut dysbiosis. By definition, prebiotics are substrates that can selectively be used by host microorganisms for beneficial effects [131]. Probiotics are live microorganisms that can exert a beneficial effect on the skin or intestinal flora by inhibiting the adhesion of pathogenic molecules to the epithelium, controlling epithelial permeability, and decreasing the release of proinflammatory cytokines and microbial peptides [132,133,134]. *Lactobacillus* and *Bifidobacterium* are well-known microorganisms used as probiotics. Clinically, beneficial effects of oral probiotics on various GI disorders, including irritable bowel syndrome, gastroenteritis, diarrhea, and inflammatory bowel disease, have been well demonstrated [134,135,136].

Various clinical studies have also confirmed that the use of probiotics is effective in controlling GI cancer [137,138,139,140]. Growing evidence suggests that probiotics can exert their action on GI cancer via their antiproliferative or pro-apoptotic properties [141,142]. Oral administration of probiotics is effective in reducing small intestinal bacterial overgrowth, which is commonly associated with GI cancer [143]. In addition, oral administration of probiotics is effective in improving GI cancer-associated symptoms among patients with gastric and colon cancers [143].

Restoring the gut microflora can improve skin conditions by indirect modulation. With regards to the skin, oral intake of probiotics has been demonstrated to be effective in improving various dermatological conditions, including atopic dermatitis, acne, and psoriasis [144,145,146].

A variety of evidence support that disturbance in the skin and/or gut microbiota is associated with the development of skin cancers. UV radiation is a potent environmental risk factor for skin cancer, and some recent studies have demonstrated a photoprotective effect of oral probiotics. Friedrich et al. [105] found that oral administration of LTA from *Lactobacillus rhamnosus* GG can overcome UVB-induced immunosuppression and decrease the growth of skin tumors in a mouse model. Intravenous administration of 6-HAP from a specific *S. epidermidis* strain was shown to have an antiproliferative effect against UV-induced skin tumors [11]. Weill et al. [104] also demonstrated that oral administration of LTA GG can delay the development of UV-induced tumor. The authors suggested that probiotics can directly modulate the cutaneous immune system and restore the homeostasis via the gut–skin axis [104]. A study by Hong et al. [147] also demonstrated that oral administration of *Bifidobacterium longum* exerts photoprotective effects on the skin of hairless mice. In another hairless mouse study, oral administration of *Lactobacillus johnsonii* offered protection against UVR-induced suppression of contact hypersensitivity [148]. It was suggested that oral probiotics helps the maintenance of skin immune homeostasis against UVR [148].

Studies have shown a beneficial effect of topical probiotics on skin disorders via a more direct effect on the skin microbiota. For instance, topical application of *Roseomonas mucosa* improved atopic dermatitis [149]. In patients with acne, 8 weeks of topical application with enterocins from *Enterococcus Faecalis* SL-5 led to clinical improvement of acne [150]. Topical application of *Lactobacillus plantarum* was shown to decrease *P. aeruginosa* colonization on skin in both in vivo and in vitro experiments [151]. In addition, topical application of *Bifidobacterium longum* lysate decreased skin inflammation, which was mediated by substance P [152]. Although no study has tested the clinical effect of topical probiotics on skin cancer to date, topical probiotics should have more direct actions on the skin microbiota in skin cancer patients. The mechanism of action of topical probiotics in skin cancer may be the modulation of the skin and intratumoral microenvironment through increased immune surveillance and suppression of chronic inflammation.

## 6. Conclusions

Recent advances in microbial research offers us a wider understanding on the pathogenesis and treatment of cancer. In this review, we looked into the dysbiosis of the skin microbiota in various skin cancers (Table 1). Activation of the skin immune system, production of microbial metabolites and toxins, barrier disruption, and UV radiation altogether may be associated with alterations of skin microbiota, leading to the initiation and progression of skin cancer, and its response to therapy. However, few studies have identified the role of the skin microbiota in the activation of the direct mutagenic pathway and the modulation of oncogenic pathways. Therefore, additional studies are needed to elucidate the role of the skin microbiome in skin cancer.

It is widely understood that the gut microbiota modulates the skin and such skin–gut axis may be involved in the skin cancer pathogenesis and treatment responses. However, the likely indirect role of the gut microbiota placed on skin cancer development should be more precisely elucidated in the future. Unlike other solid organ cancers, UVR and skin barrier disruption are pivotal factors in the initiation and progression of skin cancer. Therefore, combining the effect of these risk factors and skin and gut microbiota will more clearly declare the role of microbiota in skin cancer.

The study of the human microbiota in skin cancer is currently ongoing. If research continues along with future advances in microbiomics, we are positive that complex host–microbial interaction and its role in skin cancer will be better understood in the future. Such studies can lead to the early detection, preventive measures, and supplemental therapy for skin cancer.

## Figures and Tables

**Table 1:** Various skin microbiota associated with skin cancers and their proposed mechanisms.

Skin Microbiota	Sample Collection	Proposed Mechanisms
**Squamous cell carcinoma**		
Increased abundance and/or associated with carcinogenesis		
*Staphylococcus aureus* [5,6,7]	Human, skin biopsy, and swab [5] Human, skin swab [6] Human, skin biopsy [7]	Promotes chronic inflammation Associated with increased expression of hBD-2, which causes in the proliferation of tumor cells Results from barrier disruption
Beta HPV types [27,28]	Mouse model, skin biopsy [27] Human, skin biopsy [28]	Act as cocarcinogens, promoting cellular damage under UV irradiation but are not required for the maintenance of SCC
Merkel cell polyomavirus [31]	Human, skin biopsy, blood, mouthwash [31]	Not suggested
Decreased abundance and/or associated with anti-tumor action		
*Cutibacterium* spp.[6]	Human, skin swab	Altered metabolism in SCC might inhibit the growth of *Cutibacterium* and induce the growth of *Staphylococcus aureus*
*Malassezia* spp. [6]	Human, skin swab	Results from skin barrier disruption and decreased sebum availability in SCC Inhibits the growth of *S. aureus* biofilm formation
*Staphylococcus epidermidis* [11]		*S. epidermidis*-derived 6-HAP suppress the synthesis of DNA and exert an antiproliferative effect on tumor cells
**Malignant melanoma**		
Increased abundance and/or associated with carcinogenesis		
*Corynebacterium* spp. [34] *Staphylococcus epidermidis* [38] *Fusobacterium nucleatum* [40] High-risk mucosal HPVs [45]	Human, skin swab Pig, skin scrape Human, skin biopsy	Enhance IL-17-dependent pathway Enhances survival of melanocytes via upregulation of TRAF1, CASP14, CASP5, and TP73 during UVB irradiation Inhibits NK cells cytotoxicity through interaction with Fap2 and TIGIT May serve as a cofactor in the development of MM
Decreased abundance and/or associated with anti-tumor action		
*Cutibacterium acnes* [37,38]		Induces Th1 cytokines including −12, TNF-α, and IFN-γ Promotes apoptosis, enhancing the secretion of coproporphyrins, and upregulating TNFα
*Staphylococcus epidermidis* [11]		*S. epidermidis*-derived 6-HAP inhibits the growth of B16F10 melanoma cell lines
**Cutaneous T cell lymphoma** Increased abundance and/or associated with carcinogenesis		
*Staphylococcus aureus* [56,57] *Staphylococcus argenteus* [61]	Human, skin biopsy, and swab [57] Human, skin swab [56]	Bacterial superantigenic stimuli (TSST-1) and staphylococcus enterotoxin A activate the STAT3 pathway A possible pathogenic role of alpha-hemolysin exotoxin from *S. argenteus*

Abbreviation: hBD-2, human beta-defensin-2; HPV, human papillomavirus; 6-HAP, 6-N-hydroxyaminopurin; IFN, interferon; SCC, squamous cell carcinoma; TNF, tumor necrosis factor; TSST, toxic shock syndrome toxin -1.

## Abbreviations

6-HAP	6-N-hydroxyaminopurin
AK	actinic keratosis
AMP	antimicrobial peptide
BCC	basal cell carcinoma
CI	confidence interval
CRC	colorectal cancer
CTCL	cutaneous T cell lymphoma
DAMPs	damage-associated molecular patterns
DCA	deoxycholic acid
DNA	deoxyribonucleic acid
EPI	Envoplakin, periplakin and involucrin
GI	gastrointestinal
hBD	human beta defensin
HERV	human endogenous retrovirus
HPV	human papillomavirus
HTLV	human T cell lymphotropic virus
ICI	immune checkpoint inhibitors
IL	interleukin
INF-γ	interferon gamma
LTA	lipoteichoic acid
MCC	Merkel cell carcinoma
MCPyV	Merkel cell polyomavirus
MF	mycosis fungoides
MM	malignant melanoma
NMSC	non-melanoma skin cancer
PAMPs	pathogen-associated molecular patterns
PCR	polymerase chain reaction
PD	programmed cell death
PRRs	pattern recognition receptors
PSM	phenol-soluble modulin
SCC	squamous cell carcinoma
SCFAs	short chain fatty acids
SS	Sezary syndrome
TLR	Toll-like receptor
TME	tumor microenvironment
TNF-α	tumor necrosis factor alpha
UV	ultraviolet
UVR	ultraviolet radiation
